# Journalism

**Published:** 1909-08-14

**Authors:** 


### The Practitioner's Relaxations.

### JOURNALISM.

An important feature of the events of the last fifteen years has been the growth, at a continually accelerating rate, of journalism. Time was when newspapers merely supplied a limited *clientèle* with information on current affairs, apportioning space to subjects according to their relative importance. For good or evil this has since been changed, first by Mr. Stead and then by the Harmsworths and others, who, coming at a time when the Education Act had greatly enlarged the literate class, made it their business to write up topics entertainingly. The "new journalism" thus started by them has, as everybody knows, achieved a great popular success, one of the minor results having been to increase hugely the number of occasional contributors to the Press, or, in journalistic slang, "free lances." The great bulk of the urban population having now to be daily informed, amused, and instructed through the medium of print, there is naturally an opening for anyone who can help to provide suitable mental provender. Not the least of the qualifications for doing so is the possession of special experience, which, indeed, may be of almost any nature whatsoever. So long as his subject has a fair amount of human interest, anybody with the capacity (and, of course, the taste) for popular exposition can add to his income by retailing his knowledge realistically or romantically at so much per thousand words. And the medical man, although he has not quite seen it yet, may do so too.

### A Field for the General Practitioner.

It is an easy proposition to defend that in such a case he need infringe the rules neither of good taste nor of professional etiquette. If his name appear but on the back of the manuscript, and if only such subject matter be taken as is suitable for reputable journalistic treatment, the most critically minded in respect of medical ethics can raise no objection. Further, the informative medical article which is likely to form a considerable part of such output should be productive of nothing but good to all concerned, mainly because enlightenment of the public mind on such matters must, if the temptation to flavour them with idealistic mental placebos be resisted, help to bring about a state of public opinion which is at present sadly lacking. The author himself, too, may get some additional culture, although of a different kind.

### Good and Bad Writing.

Unfortunately, our medical journals often contain contributions which, even after editorial furbishing, fall in the matter of style far below the lay journalistic standard. Writing, indeed, is much harder than is suspected. To begin with, distinct natural aptitude is required. A woman journalist lately remarked that writing was like flirting: those who would do it would practise themselves in the art without waiting to be told how, while nobody could teach the others. This is overstating the case. Force of diction and power of happy expression are out of reach of most, but everyone should be able to learn to write grammatically, and, provided the meaning attempted is not too complex, clearly. Failing the peculiar faculty of a Dryden or a Scott, who seldom revised what they wrote, an immensity of patient practice is required. With regard to this the aphorism just quoted is true enough; only those with aptitude will have the patience. All need instruction, because all beginners tumble into the same pitfalls. One of the commonest of these is to think that a good style implies high-flown diction. The usual illustration of this error is to advise comparison of a piece of Milton's or of Ruskin's prose with one by Cowper or Swift. Were these passages read to a popular audience there is no doubt that the general vote would place the first two far above the others. Yet in reality Ruskin's rhetorical grace is a much less vital element of good prose than the strength and directness of Swift, and much easier to imitate. One should try to set out one's meaning so that it cannot fail to impress itself strongly on the mind of the most careless reader. To invert a well-known maxim, it may be said that the object of ordinary good writing is to make easy reading.

### The Importance of Being Thorough.

To successful accomplishment of this hard task there are many pre-requisites. Hackneyed phrases must be avoided because by over-use their power of conveying sharp impressions has gone. In the search for synonyms the thesaurus is not to be despised as a mechanical aid, although the literary aspirant should try to acquire a large vocabulary of his own by study of good authors and of the dictionary, especially the etymological dictionary. Precision of style comes from writing just the word called for by the meaning, and no other. "Concise" and "succinct," for instance, do not mean the same thing, and therefore are not to be used indifferently. Having formed one's sentences with care for variety and got them to follow on one another connectedly and with the right punctuation, one should revise repeatedly with a view mainly to compression. Redundant words are a great hindrance to what Mr. Shaw calls "effectiveness of assertion." Probably a good many commas too will have to come out. Newman's *Apologia* and the schoolboy's essay are both peppered with them, but in the many intervening grades of prose composition it is well to limit their use to places where the voice naturally pauses. Idiom is valuable, but it should never lead into colloquialisms. In short, infinite pains need to be taken over whatever is written, since thus only can the writer get the practice necessary to develop his literary powers to their utmost, which in turn means, of course, the opportunity of better-paid work. Many good people look down on the popular article, whom it would greatly benefit as an exercise in composition.

